# Bilateral Ectopic Hypoplastic Uteri Attached to Bilateral Pelvic Sidewalls in a 21-Year-Old Patient with Primary Amenorrhea: The First Published Report

**DOI:** 10.1155/2013/450165

**Published:** 2013-10-31

**Authors:** Ahmed Nazer, Ahmed Abu-Zaid, Osama AlOmar, Hany Salem, Ayman Azzam, Ismail A. Al-Badawi

**Affiliations:** ^1^Department of Obstetrics and Gynecology, King Faisal Specialist Hospital and Research Center (KFSH&RC), P.O. Box 3354, Riyadh 11211, Saudi Arabia; ^2^College of Medicine, Alfaisal University, P.O. Box 50927, Riyadh 11533, Saudi Arabia; ^3^Department of General Surgery, Faculty of Medicine, Alexandria University, Alexandria 21526, Egypt

## Abstract

Müllerian duct anomalies (MDAs) encompass a group of anatomical malformations resulting from defective development, fusion, migration, or resorption of Müllerian (paramesonephric) ducts during embryonic life. Herein, we report the first case of an exceedingly uncommon MDA (bilateral ectopic hypoplastic uteri attached to bilateral pelvic sidewalls) in a 21-year-old woman who was referred to our tertiary care center as a case of primary amenorrhea for workup and further management.

## 1. Introduction

Müllerian duct anomalies (MDAs) include a collection of anatomical malformations resulting from defective development, fusion, migration, or resorption of Müllerian (paramesonephric) ducts during embryonic life [1]. Clinical diagnosis of MDAs is largely delayed or missed as a large proportion of these patients are symptom-free [2]. Imaging is greatly helpful in identifying and categorizing the MDAs so that the most appropriate management plan is offered [3, 4]. The most popular classification system of MDAs has been formulated by the American Society of Reproductive Medicine (ASRM) and includes seven major categories [3]. Herein, we report the first case of an exceedingly uncommon MDA (bilateral ectopic hypoplastic uteri attached to bilateral pelvic sidewalls) in a 21-year-old woman who was referred to our tertiary care center as a case of primary amenorrhea for workup and further management.

## 2. Case Report

A 21-year-old Saudi lady, married for two months, was referred to our tertiary care center as a case of primary amenorrhea for work up and further management. Patient denied any history of oral contraceptive pill intake, strenuous exercise, cyclical abdominal pain, genitourinary, or gastrointestinal symptoms. Family history of similar clinical condition was negative. Family history of obstetric, gynecological, and endocrinological problems was negative too. Past medical history and past surgical history were unremarkable. 

On physical examination, height and weight of patient were 150 cm and 58 kg, respectively. Body mass index (BMI) was 25.8. Patient had normal hair distribution, normal breast development, and normally appearing external genitalia. Bimanual examination showed a severely hypoplastic vagina of 1 cm in length. Moreover, cervix and uterus could not be palpated.

Laboratory investigations including complete blood count, hepatic, renal, bone, and full hormonal profiles (including LH, FSH, estrogen, and testosterone) were within normal ranges. 

**Figure 1:**
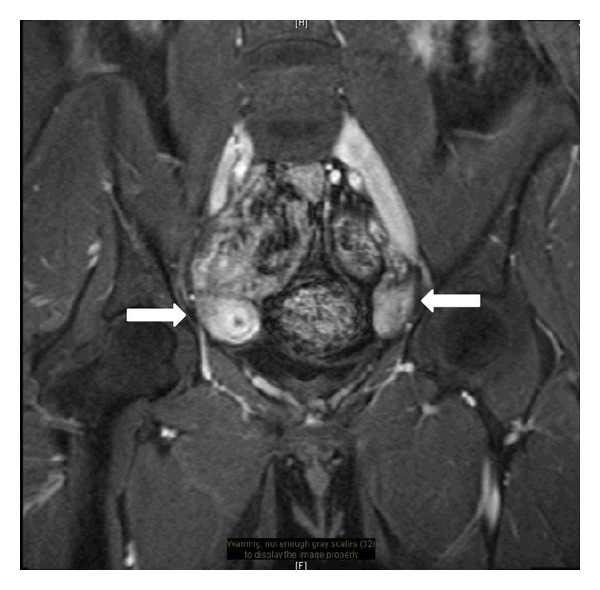
Coronal magnetic resonance imaging (MRI) scan showing two masses seen at bilateral pelvic sidewalls demonstrating enhancement patterns suggestive of possible uterine tissue (arrows). The right and left masses measured 3.2 × 1.9 cm and 2.9 × 1.6 cm in the maximum dimensions, respectively.

Magnetic resonance imaging (MRI) showed severe hypoplasia of the lower one-third of vagina (1 cm in length) and agenesis of the upper two-thirds of vagina and cervix. Uterus was not identified in the expected location. There were two masses seen at both pelvic sidewalls demonstrating enhancement patterns suggestive of possible uterine tissue. The right and left masses measured 3.2 × 1.9 cm and 2.9 × 1.6 cm in the maximum dimensions, respectively (Figure 1). The bilateral fallopian tubes and ovaries were grossly unremarkable. No obvious renal anomaly was identified. The following radiological impression was made: bilateral ectopic hypoplastic uteri (attached to bilateral pelvic sidewalls) with severe hypoplasia of the lower one-third of vagina and agenesis of the upper two-thirds of vagina and cervix. Patient was advised for genetic karyotyping and exploratory laparoscopy.

Genetic karyotyping was done and showed a normal female karyotype (46, XX).

**Figure 2:**
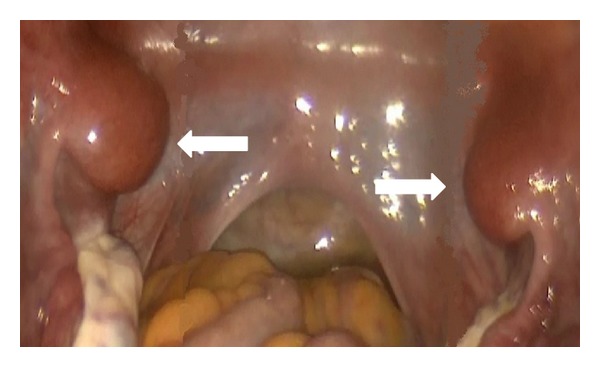
Pelvic exploratory laparoscopy showing bilateral ectopic rudimentary (hypoplastic) uteri measuring approximately 2 × 2 cm and attached to right and left pelvic sidewalls (arrows) with empty pelvis.

Exploratory laparoscopy was done and showed bilateral ectopic rudimentary (hypoplastic) uteri measuring approximately 2 × 2 cm and attached to right and left abdominal sidewalls with normal bilateral fallopian tubes and ovaries and empty pelvis (Figure 2). Cervix and upper two-thirds of vagina were not identified.

The laparoscopic findings were explained to patient. The patient was informed about impossibility of conception (getting pregnant) in the current settings. Moreover, patient was offered the option of cosmetic vaginoplasty for the severe hypoplasia of the lower one-third of vagina and agenesis of the upper two-thirds of vagina. Patient accepted the vaginoplasty procedure and it was done successfully without complications.

## 3. Discussion

Müllerian duct anomalies (MDAs) encompass a heterogeneous group of anatomical malformations arising from defective process in one or more of the following phases of embryonic development. These phases include (1) abnormal organogenesis, (2) defective vertical/lateral migration and fusion, and (3) septal resorption failure of Müllerian ducts during embryogenesis [1]. 

Exact incidence and prevalence rates of MDAs are largely difficult to estimate. This can be broadly attributed to a variety of reasons. Such reasons include (1) majority of patients are asymptomatic, (2) evaluation of highly diverse patient populations, (3) utilization of quite nonstandardized classification schemes, and (4) wide discrepancy in use of diagnostic modalities with subsequent attainment of variably diverse diagnostic results [2, 5]. Grimbizis and colleagues [6] reported that prevalence of MDAs was approximately 4.3% in general population and/or fertile women, 3.5% in infertile women, and 13% in women with obstetric complications (particularly recurrent pregnancy losses).

Clinical diagnosis of MDAs is most often delayed or missed as vast majority of patients are asymptomatic [2]. Other plausible reasons for delayed or missed clinical diagnosis include (1) insignificant obstetric/gynecological physical examination findings, (2) normally functioning ovaries, (3) normally appearing age-appropriate external genitalia, and (4) minor anomalies that do not cause any menstrual, coital, or obstetric adverse events. However, diagnosis of MDAs may be established (1) during assessment of patients with history of amenorrhea, dysmenorrhea, chronic pelvic pain, infertility, or recurrent pregnancy losses, (2) when complications occur during periods of antenatal care or obstetric labor, or (3) when genital outflow tract obstruction happens and patients present with abdominal space-occupying masses or fluid-filled collections [5, 7]. In addition, it must be realized that higher incidences of primary amenorrhea, infertility, spontaneous miscarriages, recurrent pregnancy losses, fetal intrauterine growth restriction, fetal malposition, preterm birth, and retained placenta have been well documented in patients with MDAs [3]. In our case, primary amenorrhea was the main presenting symptom. 

Imaging plays vital roles in identifying and categorizing the MDAs, so that the most appropriate management is offered [3, 4]. Owing to intricacy of presentations, establishing accurate diagnosis of MDAs usually demands utilization of more than one diagnostic imaging modality in approximately 62% of the cases [8, 9]. Such imaging modalities generally include hysterosalpingography (HSG), two-dimensional (2D) ultrasonography, and magnetic resonance imaging (MRI).

HSG has been traditionally the diagnostic imaging modality of choice used to assess the cervical canal, uterine cavity, and fallopian tubes. However, its use is greatly limited in patients who are still virgins [10]. Moreover, its specificity in diagnosing MDAs ranges from 6% to 60%, and it is highly dependent on the technician's expertise and type of anomaly evaluated [10, 11].

Conventional 2D ultrasonography facilitates a much more thorough analysis of the cervical canal, endometrium, and uterine cavity. Its specificity ranges from 85% to 92% [12–14]. It is characterized by being rapid, easily accessible, cost-effective, and radiation-free [4]. However, its use is limited by being operator-dependant and highly affected by fat layers and overlying bowel gas [4]. Lately, three-dimensional (3D) ultrasonography has been proved to offer higher specificity than 2D ultrasonography with relatively comparable results to MRI at evaluating MDAs [15]. It is anticipated that 3D ultrasonography has the prospective of emerging as the imaging modality of choice for diagnosing MDAs [15].

However, at the present time, MRI remains the gold standard imaging modality of preference for diagnosing MDAs [16]. It possesses very high specificity ranging from 96% to 100% in diagnosing MDAs [11] and has been shown to accurately characterize (categorize) the MDAs subtypes [16]. Moreover, it advantageously lacks ionizing radiation and offers optimal delineation of the intrauterine anatomy and extrauterine anatomy [16]. In our case, we opted to use MRI as the first-line imaging modality due to the above-mentioned advantages and direct accessibility to MRI without the need to utilize HSG or 2D/3D ultrasonography.

The most commonly used classification system of MDAs is that established by the American Society of Reproductive Medicine (ASRM) based on anatomical manifestations [3]. However, it must be noted that this classification system does not cover all MDAs and some anomalies will not fit totally into one of the classification system categories. In such conditions, as in our case, it is highly recommended to precisely depict the elements of anomalies rather than randomly assigning the anomalies to a classification system category that does not completely represent it.

In our case, as revealed by MRI, patient had bilateral ectopic hypoplastic uteri (attached to the pelvic sidewalls) with bilateral normal ovaries and fallopian tubes. Furthermore, patient had agenesis of cervix and upper two-thirds of vagina in addition to severe hypoplasia of lower one-third of vagina (approximately 1 cm in length). The bilateral ectopic locations of hypoplastic uteri attached to the pelvic sidewalls have never been reported before in the literature or described in the ASRM classification system of MDAs. Therefore, it was difficult to place our case anomaly findings into one of the ASRM classification system categories. Moreover, we, possibly, propose incorporating our atypical MDA findings as a new category into the ASRM classification system of MDAs. 

It is very vital to accurately classify MDAs in order to undertake the most appropriate management plan [3, 4]. In our case, the impossibility of conception (pregnancy) was explained to the patient. Moreover, our patient did not experience cyclical (menstrual) pain, and accordingly we concluded that there were no many endometrial layers in both hypoplastic uteri. Thus, we decided not to surgically remove the hypoplastic uteri as it would be of no benefit and rather might result in unnecessary major complications, particularly ureters and uterine vessels. Patient was counseled for cosmetic and conservative vaginoplasty only.

Renal malformations happen in nearly 30% of patients with MDAs and renal agenesis is the most frequently encountered malformation (67%) [17]. Other renal malformations associated with MDAs comprise renal dysplasia, ectopic kidney, horseshoe kidney, and duplicated collecting systems [17]. In our case, MRI did not identify any renal anomalies.
